# Comparative Genomic Analysis Reveals Evolutionary Features of *Glugea plecoglossi*, a Fish-Infecting Microsporidian Parasite of Ayu (*Plecoglossus altivelis*)

**DOI:** 10.3390/biology15201848

**Published:** 2026-10-15

**Authors:** Lijun Duan, Yiming Zheng, Qianjin Zhou, Yinghao Zhou, Maocang Yan, Yuanyi Liu, Ye Tao, Jiong Chen

**Affiliations:** 1 Laboratory of Biochemistry and Molecular Biology, School of Marine Sciences, Ningbo University, Ningbo 315211, China; duanjie1207@163.com (L.D.); zhengyiming0619@163.com (Y.Z.); zyh18868251229@163.com (Y.Z.); liuyuanyi0504@163.com (Y.L.); 2 Key Laboratory of Aquacultural Biotechnology of Ministry of Education, Ningbo University, Ningbo 315211, China; 3 Ningbo Haishu District Animal Husbandry and Veterinary Medicine Technical Management Service Station, Ningbo 315000, China; 4 Zhejiang Key Laboratory of Coastal Biological Germplasm Resources Conservation and Utilization, Zhejiang Mariculture Research Institute, Wenzhou 325000, China; yanmaocang@126.com; 5 Shanghai BIOZERON Biotechnology Co., Ltd., Shanghai 201800, China; taoye@biozeron.com

**Keywords:** *Glugea plecoglossi*, microsporidia, comparative genomics, genome evolution, functional repertoire

## Abstract

Microsporidia are obligate intracellular parasites with extensively reduced genomes, yet substantial genomic variation occurs among lineages. *Glugea plecoglossi* infects ayu (*Plecoglossus altivelis*) and forms conspicuous xenomas, but its genome characteristics and evolutionary position remain poorly resolved. We generated a genome assembly of *G. plecoglossi* and compared it with 42 additional microsporidian taxa using phylogenomics, functional profiling, repeat analysis, and multi-copy orthogroup analysis. The 16.71 Mb assembly comprised 59 scaffolds, with a scaffold N50 of 557.5 kb and 78.5% complete BUSCOs, and contained 4331 predicted protein-coding gene loci. Phylogenomic analysis recovered *G. plecoglossi* as the sister taxon to *Pseudoloma neurophilia*. Compared with closely related taxa, *G. plecoglossi* had a substantially larger genome and more predicted protein-coding genes. Major Gene Ontology profiles were broadly similar across taxa, whereas metabolism-related profiles varied considerably. Among 11 genomes, repetitive-sequence composition differed markedly, with *G. plecoglossi* showing particularly high long terminal repeat coverage and substantial DNA-transposon coverage. The retained multi-copy orthogroups showed highly heterogeneous member distributions across taxa, with relatively high member numbers in *G. plecoglossi* restricted to a limited subset. These findings highlight genomic and functional variation among microsporidian lineages and provide a comparative genomic resource for future studies of fish-infecting microsporidia.

## Simple Summary

Microsporidia are microscopic parasites that can grow only inside host cells. Many species have extremely small genomes, meaning that they have lost much of their genetic material during adaptation to this way of life, but genome size and gene content can differ greatly among evolutionary groups. *Glugea plecoglossi* infects ayu and forms large parasite-containing structures called xenomas, yet little is known about its genome or evolutionary relationships. We sequenced the genome of *G. plecoglossi* and compared it with those of 42 other microsporidian groups. Its genome was substantially larger and contained more genes predicted to encode proteins than those of its closest relatives. Despite this difference, the broad types of protein functions were generally similar across microsporidia, whereas metabolism-related functions, repetitive DNA, and several groups of related proteins varied markedly among evolutionary groups. These results show that microsporidian genome evolution combines widely conserved features with lineage-specific changes rather than following a uniform pattern of genome reduction. This study expands genomic resources for fish-infecting microsporidia and provides a basis for future research on parasite evolution, host interactions, and microsporidian infections in aquatic animals.

## 1. Introduction

Microsporidia are a diverse group of highly specialized obligate intracellular eukaryotic parasites that infect a broad range of animal hosts. Their life cycles are characterized by environmentally resistant spores containing a unique invasion apparatus, the polar tube, which rapidly delivers the infective sporoplasm into host cells. The transition to obligate intracellular parasitism has been accompanied by extensive cellular and genomic reduction. Canonical microsporidia possess highly reduced mitochondrial remnants, termed mitosomes, and depend heavily on host-derived energy and metabolites for intracellular growth and reproduction [1,2,3].

Genome reduction and compaction are among the most prominent features of microsporidian evolution. The approximately 2.9 Mb genome of *Encephalitozoon cuniculi*, for example, exhibits extensive gene loss, short intergenic regions, and a highly compact genomic architecture [4]. However, increasing genomic sampling has shown that microsporidian evolution cannot be described simply as progressive genome reduction. Genome sizes differ markedly among lineages, and larger genomes may contain expanded non-coding regions, abundant repetitive sequences and transposable elements, or lineage-specific protein families [1,5,6,7]. Comparative genomic analyses have therefore revealed a dynamic evolutionary pattern in which ancestral gene loss and genome compaction coexist with lineage-specific changes in genome structure and protein content.

Metabolic remodeling represents another major consequence of intracellular adaptation. Microsporidia have lost numerous enzymes involved in biosynthetic and energy-generating pathways, while retaining a restricted set of functions required for essential cellular processes and intracellular survival [1,7]. Importantly, functional reduction is not uniform across the phylum. Core cellular processes remain broadly conserved across many lineages, whereas individual metabolic and regulatory modules have undergone independent losses or differential retention [6,8]. For example, *Enterocytozoon bieneusi* has lost much of its core carbon metabolism, and further decay of glycolysis has been documented across the Enterocytozoonidae [9,10]. These observations indicate that comparative analyses within a phylogenetic framework are required to distinguish broadly conserved features of microsporidia from lineage-specific patterns of functional retention and loss.

Microsporidian infections are also important in aquatic animals. *Enterocytozoon hepatopenaei* is a major pathogen of cultured penaeid shrimp and has become an important model for investigating the genomic characteristics of aquatic microsporidia [11,12]. Several fish-infecting microsporidia, including species of *Loma* and *Glugea*, induce pronounced hypertrophy of infected host cells and the formation of xenomas, which provide a distinctive intracellular environment for parasite development [13,14]. *Glugea plecoglossi* is a microsporidian parasite of ayu (*Plecoglossus altivelis*) that forms conspicuous xenomas in infected tissues [15]. Previous studies of this parasite have primarily addressed its occurrence, morphology, pathology, and molecular detection, whereas its genome organization, functional repertoire, and evolutionary position relative to other microsporidia remain poorly characterized.

In this study, we generated a genome assembly of *G. plecoglossi* from parasites isolated from naturally infected ayu and placed this genome within a comparative framework comprising 42 additional microsporidian taxa. We examined genome size, protein-coding capacity, genome completeness, and selected morphological and molecular characteristics across the phylogeny. We further compared proteome-wide Gene Ontology profiles, metabolism-related functional categories, repetitive-sequence composition, and the distribution of multi-copy orthogroups. This integrated analysis was designed to establish a genomic resource for *G. plecoglossi* and to determine how its genomic and functional characteristics relate to the broader patterns of reduction, conservation, and lineage-specific diversification observed across microsporidia.

## 2. Materials and Methods

### 2.1. Sample Collection, Parasite Purification, and Identification

*Glugea plecoglossi* was obtained from naturally infected ayu (*Plecoglossus altivelis*) collected from an aquaculture farm in Ningbo, Zhejiang Province, China. The fish were not subjected to experimental infection or other experimental interventions and were euthanized with an overdose of eugenol before dissection. According to the institutional animal ethics review policy of Ningbo University, this study fell outside the formal administrative review scope of the Animal Ethics and Welfare Committee of Ningbo University. Conspicuous white to milky xenomas were isolated from affected tissues and maintained in sterile phosphate-buffered saline (PBS) at 4 °C until processing. Xenomas collected from multiple naturally infected fish were pooled to obtain sufficient parasite material for whole-genome sequencing.

The isolated xenomas were washed with sterile PBS and gently disrupted using sterile syringe needles to release the spores. The suspension was passed through a 100 μm cell strainer to remove large tissue debris, concentrated by centrifugation, and further purified by Percoll density-gradient centrifugation. Purified spores were washed with PBS and examined microscopically. Parasite identity was further confirmed by PCR amplification of a 981 bp fragment of the β-tubulin gene using previously described *G. plecoglossi*-specific primers [16], followed by Sanger sequencing. Confirmed parasite material was used for genomic DNA extraction and whole-genome sequencing.

### 2.2. Genomic DNA Extraction and Genome Sequencing

Genomic DNA was extracted from purified *G. plecoglossi* spores using the E.Z.N.A.^®^ Tissue DNA Kit (Omega Bio-tek, Norcross, GA, USA) according to the manufacturer’s instructions. DNA concentration and purity were assessed using a NanoDrop spectrophotometer (Thermo Fisher Scientific, Waltham, MA, USA), and DNA integrity was evaluated by agarose gel electrophoresis.

A combination of short- and long-read sequencing was used for genome assembly. For short-read sequencing, libraries were prepared using the Illumina TruSeq^TM^ Nano DNA Sample Prep Kit (Illumina, San Diego, CA, USA) and sequenced on an Illumina NovaSeq 6000 platform to generate 2 × 150 bp paired-end reads. Long-read libraries were prepared using the CycloneSEQ 24 Barcode Library Preparation Kit (MGI Tech Co., Ltd., Shenzhen, China) and sequenced on the CycloneSEQ platform.

### 2.3. Sequencing Quality Control and Genome Survey

Illumina reads were quality filtered using Trimmomatic v0.39 [17]. Adapter-contaminated sequences and low-quality bases were removed, bases with Phred quality scores below Q20 were trimmed, and reads containing >10% undetermined bases or shorter than 75 bp after trimming were discarded. The resulting clean reads were used for genome survey analysis and subsequent assembly.

Genome characteristics were estimated from the Illumina clean reads using 21-mer frequency analysis implemented with the assembly.kmer module of the JCVI package. The 21-mer depth distribution was used to estimate genome size, repeat content, and the model-derived sequence-variation parameter. Because the sequencing material comprised parasites pooled from multiple naturally infected fish, this parameter was interpreted as within-sample sequence variation rather than as heterozygosity of a single parasite genome.

### 2.4. Genome Assembly and Quality Assessment

The *G. plecoglossi* genome was assembled de novo using both Illumina short reads and CycloneSEQ long reads. Hybrid assembly was performed with MaSuRCA v4.0.8 [18], while an independent long-read assembly was generated with Canu v2.1.1 [19]. The two assemblies were compared using MUMmer v4.0 [20], which showed a high degree of collinearity and no substantial structural differences. The Canu assembly had a higher N50, fewer scaffolds, and higher BUSCO completeness than the MaSuRCA assembly and was therefore selected as the final assembly. The selected assembly was polished first with Racon using the long reads [21], followed by base-level correction with Illumina reads using Pilon [22].

Assembly statistics, including total assembly length, scaffold number, N50, N90, and GC content, were calculated from the final assembly. Illumina clean reads were mapped back to the assembly using BWA v0.7.15 [23], and the resulting alignments were processed with SAMtools v1.12 [24] and BamDeal v0.22 to obtain sequencing-depth and genome-coverage information. GC content and sequencing depth were jointly examined to assess the compositional distribution of assembled sequences.

Genome completeness was evaluated using BUSCO v6.0.0 [25] against the microsporidia_odb10 lineage dataset. Complete single-copy, complete duplicated, fragmented, and missing BUSCOs were quantified to assess the representation of conserved microsporidian genes in the assembly.

### 2.5. Prediction and Functional Annotation of Coding and Non-Coding Genes

Protein-coding genes were predicted using a combined ab initio and homology-based strategy. Because no genome from another *Glugea* species was available in the NCBI database, AUGUSTUS v3.4.0 [26] was trained using gene information from 43 microsporidian genomes and used for ab initio prediction. For homology-based prediction, protein sequences from the same 43 microsporidian genomes were used as reference evidence. The ab initio and homology-based predictions were subsequently integrated using EVidenceModeler (EVM) [27] to generate the final consensus gene set.

Transfer RNA genes were predicted using tRNAscan-SE v2.0 [28]. Other non-coding RNA families were identified against the Rfam database [29], and ribosomal RNA genes were predicted using RNAmmer v1.2 [30].

Predicted proteins were functionally annotated against multiple public databases, including the NCBI non-redundant protein database (NR), Swiss-Prot, eggNOG/KOG, Gene Ontology (GO), the Kyoto Encyclopedia of Genes and Genomes (KEGG), and the Carbohydrate-Active enZYmes database (CAZy). Functional assignments based on Swiss-Prot, GO, and KEGG were interpreted according to the corresponding database resources [31,32,33]. A gene was considered functionally annotated when it received an assignment in at least one of the databases examined, and the overall annotation rate was calculated from the non-redundant union of annotated gene identifiers.

### 2.6. Comparative Genome Dataset and Phylogenomic Analysis

*Glugea plecoglossi* Gpl-01 and 42 publicly available microsporidian taxa were included in the comparative analysis. The comparative taxa were selected based on phylogenetic representation, host type, and the availability of genome and proteome data. Genome assemblies and available proteomes were obtained from NCBI GenBank, and assembly accessions and host information are provided in Table S4. The *G. plecoglossi* proteome was predicted as described above. Publicly available proteomes were used for 32 taxa, whereas protein-coding genes were predicted from the genome assemblies of the remaining 10 taxa using AUGUSTUS.

Orthologous groups among the 43 proteomes were inferred using OrthoFinder v2.3.8 [34], with an MCL inflation parameter of 1.5 and the MSA mode enabled, yielding 28,210 orthogroups. Protein sequences within orthogroups were aligned using MAFFT v7.490 with the --auto option [35], and poorly aligned regions were removed using trimAl v1.4rev15 with the -automated1 option [36]. Gene trees were inferred using FastTree v2.1.11 under the JTT+CAT amino-acid substitution model [37]. The species tree was inferred from orthogroup gene trees using the STAG method implemented in OrthoFinder. STAG support values indicate the proportion of gene-tree-derived species-tree estimates supporting each corresponding bipartition. The tree was visualized using iTOL v6.

### 2.7. Comparative Analysis of Genome Characteristics

Genome assembly size was calculated as the total length of all contigs or scaffolds in the corresponding genome FASTA file, and protein-coding gene number was recorded as the number of predicted protein-coding gene loci for each taxon. Genome completeness was assessed uniformly across all 43 taxa using BUSCO v6.0.0 [25] against the microsporidia_odb10 lineage dataset. The proportions of complete single-copy, complete duplicated, fragmented, and missing BUSCOs were calculated for each genome.

Ten morphological and molecular characteristics previously used in comparative analyses of microsporidia [1] were examined: flagellum, polar tube, canonical mitochondria, ATP transporter, mitochondrial complex I, clathrin, TOR signaling, RNA interference, mRNA splicing, and glycolysis. Presence or absence of each characteristic was compiled from available genome annotations and published evidence. A characteristic was scored as present when supported by explicit genomic annotation or published experimental/comparative evidence. Absence was assigned when the feature had been reported as absent or when the corresponding core components were not detected in a sufficiently complete and annotated genome.

### 2.8. Comparative Gene Ontology Analysis

To compare functional composition among the 43 microsporidian proteomes, proteins were annotated with Gene Ontology (GO) terms using the same annotation procedure described above for *G. plecoglossi*. GO assignments were summarized by Level 2 functional category, and the number of proteins assigned to each category was calculated for each taxon. Comparisons were based on raw annotation counts, and no normalization for proteome size or annotation coverage was applied. The resulting profiles were visualized as a heatmap using ggplot2 in R v3.5.1, with taxa ordered according to the STAG species tree described above.

For a focused comparison of metabolism-related functions, seven GO categories were selected: carbohydrate metabolic process; catalytic activity, acting on a tRNA (GO:0140101; catalytic activities that modify tRNA, including aminoacylation and post-transcriptional modification or processing); cellular amino acid metabolic process; generation of precursor metabolites and energy; lipid metabolic process; nucleobase-containing small molecule metabolic process; and sulfur compound metabolic process. The numbers of proteins assigned to these categories were summarized for each taxon and displayed as stacked bars in the same phylogenetic order. Because individual proteins could be assigned to more than one GO category, category counts were not mutually exclusive, and their sum does not represent the number of nonredundant metabolism-related proteins.

### 2.9. Comparative Analysis of Repetitive Sequences

To compare repetitive-sequence composition among microsporidian genomes, *G. plecoglossi* Gpl-01 and 10 additional microsporidian taxa with available genome assemblies were analyzed using a uniform repeat-annotation pipeline. De novo repeat libraries were generated separately for each genome using RepeatModeler v2.0.5 [38]. Interspersed repetitive elements were subsequently identified and classified using RepeatMasker v4.2.1 against the January 2024 release of the Dfam database. Repeat categories included long terminal repeat retrotransposons (LTRs), long interspersed nuclear elements (LINEs), short interspersed nuclear elements (SINEs), DNA transposons, rolling-circle elements (RCs), scRNA-associated repeats, and unclassified repeats. Overlapping intervals within each repeat category were merged using BEDTools v2.30.0 [39], and genomic coverage was calculated relative to the corresponding genome assembly.

Tandem repeats were identified using Tandem Repeats Finder (TRF) v4.09 [40]. Tandem repeats with repeat-unit lengths of 2–6 bp and 10–60 bp were classified as microsatellites and minisatellites, respectively. Total tandem-repeat coverage and the coverage of microsatellite and minisatellite DNA were calculated separately. Because different repeat categories may overlap and microsatellite and minisatellite DNA represent subsets of the repeats identified by TRF, the displayed category coverages were not interpreted as mutually exclusive fractions of the genome. Repeat profiles were visualized as stacked bars using ggplot2 in R v3.5.1. The comparative analysis was based on the genomic coverage of the repeat categories described above.

### 2.10. Analysis of Multi-Copy Orthogroups and Protein Features

Multi-copy orthogroups were identified from the orthogroup member-count matrix generated by OrthoFinder v2.3.8. Orthogroups containing at least 10 protein members in at least one of the 43 taxa were retained as candidate multi-copy orthogroups. Protein sequences from these orthogroups were analyzed for Pfam domains [41], signal peptides using SignalP [42], and transmembrane domains using TMHMM [43].

Orthogroups in which at least 50% of the member proteins were predicted to contain either a signal peptide or a transmembrane domain were retained for further comparison. Selected Pfam domain classes, including peptidase, zinc-finger, leucine-rich repeat (LRR), and DUF1609 domains, were additionally annotated. For visualization, the identities of these domain classes were displayed only for orthogroups containing at least two distinct Pfam domain types. Protein-member numbers were visualized as a matrix with orthogroups as rows and taxa as columns, with taxa ordered according to the OrthoFinder species-tree topology. Signal-peptide and transmembrane-domain features and the proportions of selected Pfam domains were displayed alongside the member-count matrix. The figure was generated using iTOL v6 and TBtools v2.096.

### 2.11. AI-Assisted Language Editing

During manuscript preparation, ChatGPT (OpenAI, San Francisco, CA, USA; GPT-6) was used solely to assist with English-language editing and to improve the clarity and readability of selected sections of the manuscript.

## 3. Results

### 3.1. Genome Sequencing, Assembly, and Annotation of Glugea plecoglossi

**Figure 1:**
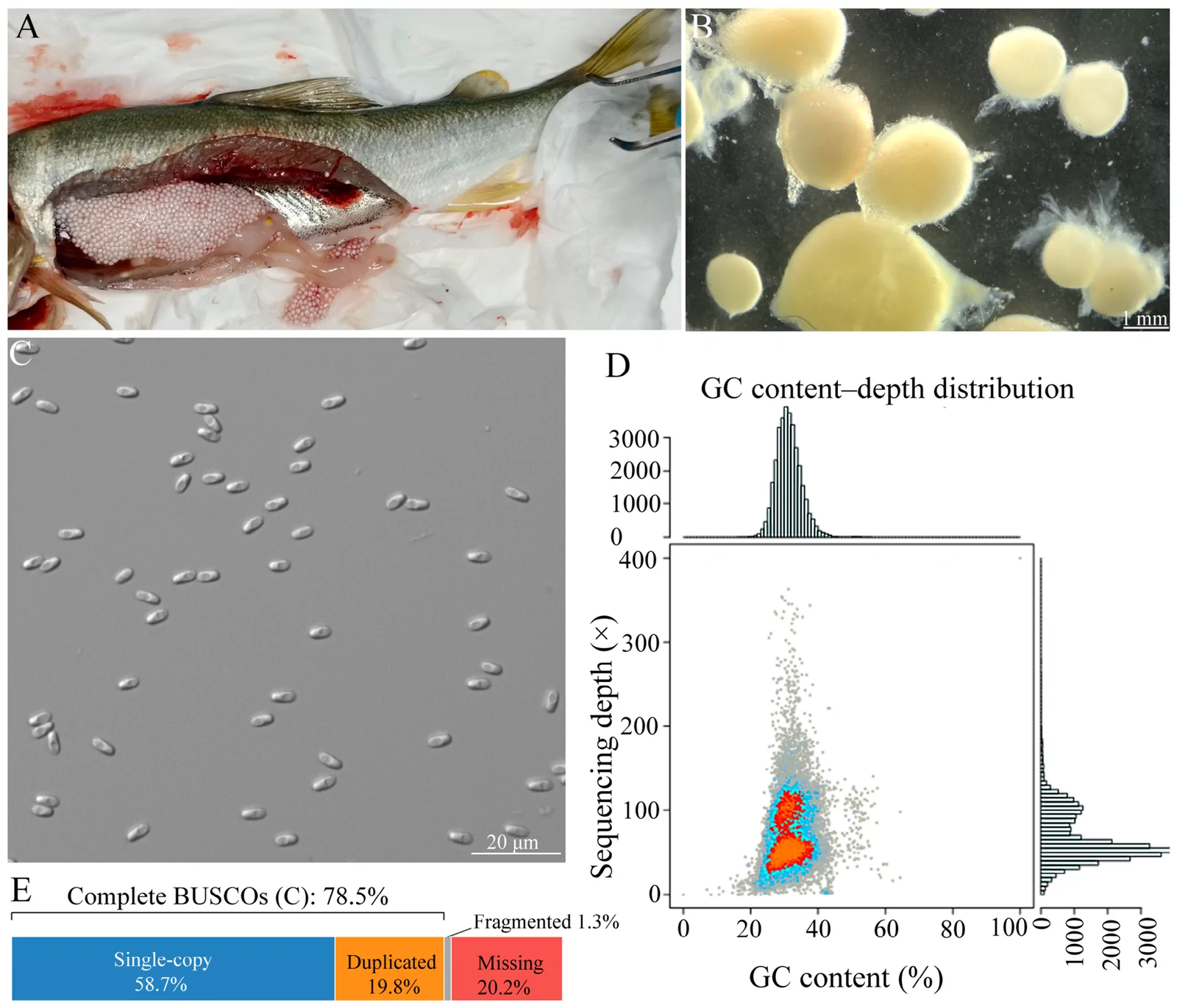
Morphological characterization of *Glugea plecoglossi* and quality assessment of its genome assembly. (**A**) Naturally infected ayu (*Plecoglossus altivelis*) showing xenomas in the abdominal cavity. (**B**) Isolated xenomas collected from infected fish. (**C**) Purified spores of *G. plecoglossi*. (**D**) GC content–depth distribution of the *G. plecoglossi* genome assembly. Scatter plot showing GC content and sequencing depth, with marginal histograms showing the corresponding distributions. Colors represent the local density of data points in the GC content–depth distribution. (**E**) BUSCO completeness assessment of the *G. plecoglossi* genome using the microsporidia_odb10 dataset. Complete BUSCOs accounted for 78.5% of the total BUSCO groups searched, including 58.7% single-copy and 19.8% duplicated BUSCOs, whereas 1.3% were fragmented and 20.2% were missing.

Naturally infected ayu exhibited numerous conspicuous white xenomas in affected tissues, from which *G. plecoglossi* spores were isolated and purified for genome sequencing (Figure 1A–C). Illumina sequencing generated 8,989,436 raw reads (1.348 Gb), of which 8,922,634 reads representing 1.338 Gb were retained after quality filtering. The clean reads had Q20 and Q30 proportions of 96.66% and 92.98%, respectively, with a GC content of 31.61% (Table S1; Figure S1A–D). Long-read sequencing generated 1,207,133 reads totaling 2.966 Gb, with a read N50 of 5479 bp, an average read length of 2457 bp, and a maximum read length of 86,397 bp (Table S1; Figure S1E).

The 21-mer survey estimated a genome size of approximately 16.1 Mb, with a characteristic coverage depth of 72×, a repeat content of 23.3%, and within-sample sequence variation of 2.68% (Table S1; Figure S1F). The 21-mer frequency distribution showed two major peaks at approximately 40× and 80× coverage, indicating substantial sequence heterogeneity in the pooled parasite material. Because the sequencing material was derived from parasites collected from multiple naturally infected fish, this variation may reflect both allelic differences within parasite genomes and genetic differences among parasite individuals and therefore cannot be interpreted solely as heterozygosity of a single genome.

The final *G. plecoglossi* genome assembly comprised 59 scaffolds with a total length of 16,711,591 bp, closely matching the genome size estimated by the 21-mer analysis. The scaffold N50 and N90 were 557,527 and 115,546 bp, respectively, with a mean scaffold length of 283,247 bp and a maximum scaffold length of 1,072,421 bp. The assembly had a GC content of 31.42% and contained no undetermined bases (Table 1). GC content–depth analysis showed that most assembled sequences were concentrated within a GC-content range of approximately 25–40% and a sequencing-depth range of approximately 50–120×, without a clearly separated high-density sequence cluster based on GC composition (Figure 1D). BUSCO analysis against the microsporidia_odb10 dataset showed that 78.5% of BUSCOs were complete, including 58.7% single-copy and 19.8% duplicated BUSCOs; 1.3% were fragmented, and 20.2% were missing (Figure 1E; Table 1).

A total of 4331 protein-coding gene loci were predicted, of which 4324 had corresponding mRNA, CDS, and protein sequences and were retained for downstream protein annotation. The mean predicted CDS length was 1262 bp, and the mean predicted protein length was approximately 420 amino acids (Table 1 and Table S2). Among the 4324 coding gene models, 3225 (74.58%) contained a single CDS segment and 1099 (25.42%) contained multiple discontinuous CDS segments, with a mean of 1.46 CDS segments per gene model (Figure S2; Table S2). In addition, 98 non-coding RNAs were annotated, comprising 70 tRNAs, 25 rRNAs, and three snRNAs (Table S2).

Functional annotation assigned 2786 of the 4324 predicted proteins (64.43%) to at least one of the databases examined (Table 1 and Table S3). NR provided the highest annotation coverage, with 2781 proteins (64.32%), followed by eggNOG/KOG with 1774 (41.03%) and Swiss-Prot with 1375 (31.80%). GO and KEGG annotations were obtained for 883 (20.42%) and 658 (15.22%) proteins, respectively, while 24 proteins (0.56%) were assigned to CAZy families. The remaining 1538 predicted proteins (35.57%) had no assignment in these databases.

### 3.2. Phylogenetic Position and Comparative Genomic Features of Glugea plecoglossi

**Figure 2:**
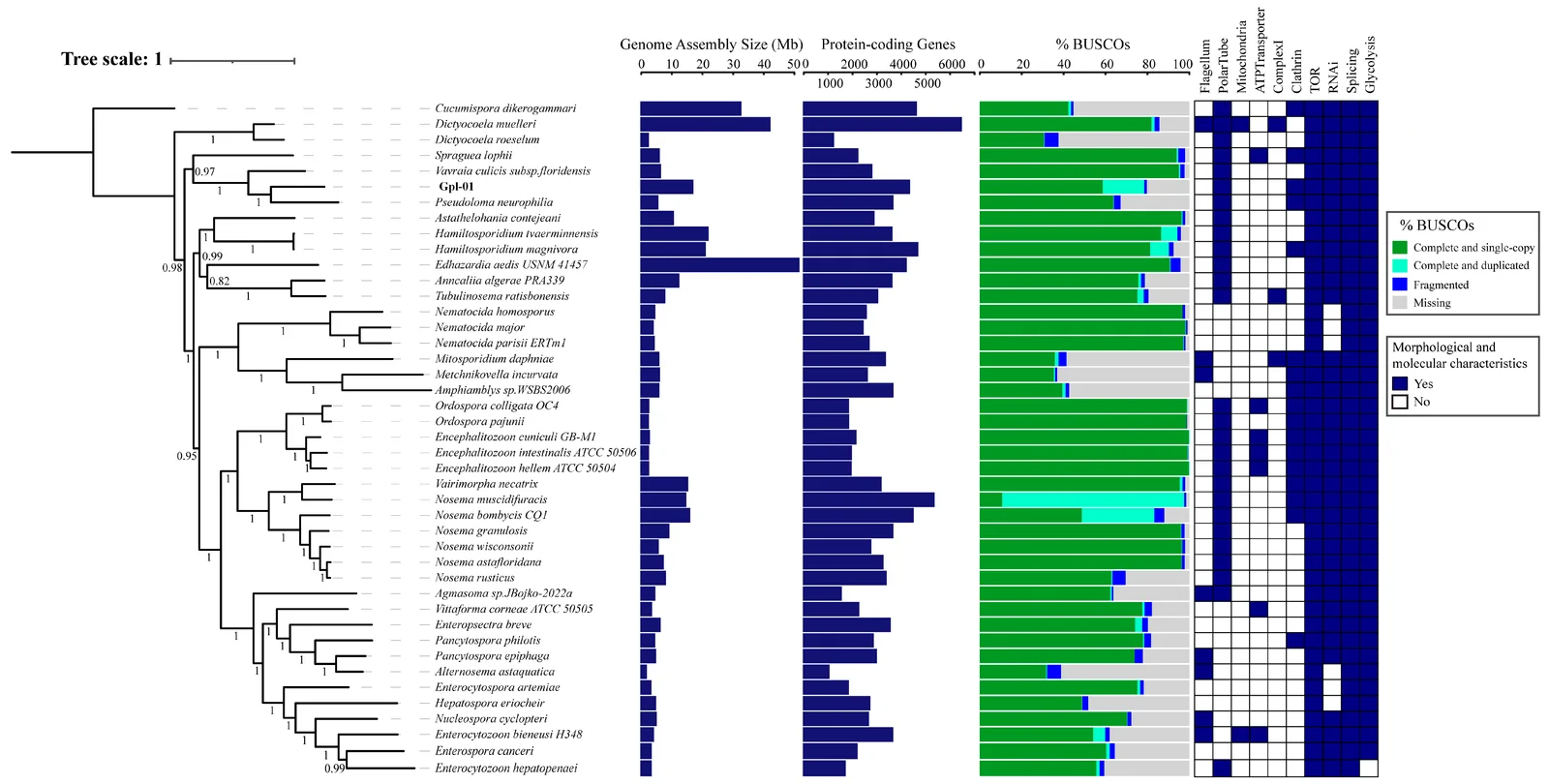
Phylogenetic position of *Glugea plecoglossi* and comparison of genomic features among microsporidia. The species tree was inferred from orthogroup-based gene trees of 43 taxa using the STAG method, with node values representing STAG support values. Horizontal bar plots show genome assembly size and the number of predicted protein-coding genes for each species. BUSCO completeness is displayed as stacked bars representing complete single-copy, complete duplicated, fragmented, and missing BUSCOs. The matrix on the right summarizes the presence or absence of 10 morphological and molecular features across the 43 microsporidian taxa, with dark blue indicating presence and white indicating absence.

The STAG species tree showed that *G. plecoglossi* Gpl-01 was most closely related to *Pseudoloma neurophilia*, with a STAG support value of 1.00 (Figure 2). The Gpl-01–*P. neurophilia* clade clustered with *Vavraia culicis* subsp. *floridensis* with a support value of 1.00, and these three taxa formed a larger clade with *Spraguea lophii* with a support value of 0.97.

Genome assembly size and predicted protein-coding gene number varied substantially among the 43 taxa (Figure 2; Table S4). Genome assembly sizes ranged from approximately 1.52 Mb in *Alternosema astaquatica* to 51.35 Mb in *Edhazardia aedis*, although the *A. astaquatica* assembly is incomplete and likely underestimates its actual genome size. The genome assemblies of *P. neurophilia* (5.25 Mb), *V. culicis* subsp. *floridensis* (6.12 Mb), and *S. lophii* (5.76 Mb) were all smaller than that of *G. plecoglossi*. *G. plecoglossi* also contained more predicted protein-coding genes than these three related taxa (Figure 2).

BUSCO profiles varied considerably among the 43 taxa, with substantial differences in the proportions of complete, fragmented, and missing BUSCOs (Figure 2). Comparison of the 10 morphological and molecular characteristics revealed heterogeneous presence/absence patterns across the taxa examined. The polar tube was broadly retained, whereas the other characteristics showed variable distributions across the phylogeny. In *G. plecoglossi*, the polar tube, TOR signaling, RNA interference, mRNA splicing, and glycolysis were scored as present, whereas flagellum, canonical mitochondria, ATP transporter, mitochondrial complex I, and clathrin were scored as absent (Figure 2).

### 3.3. Comparative GO Functional Profiles of Microsporidian Proteomes

**Figure 3:**
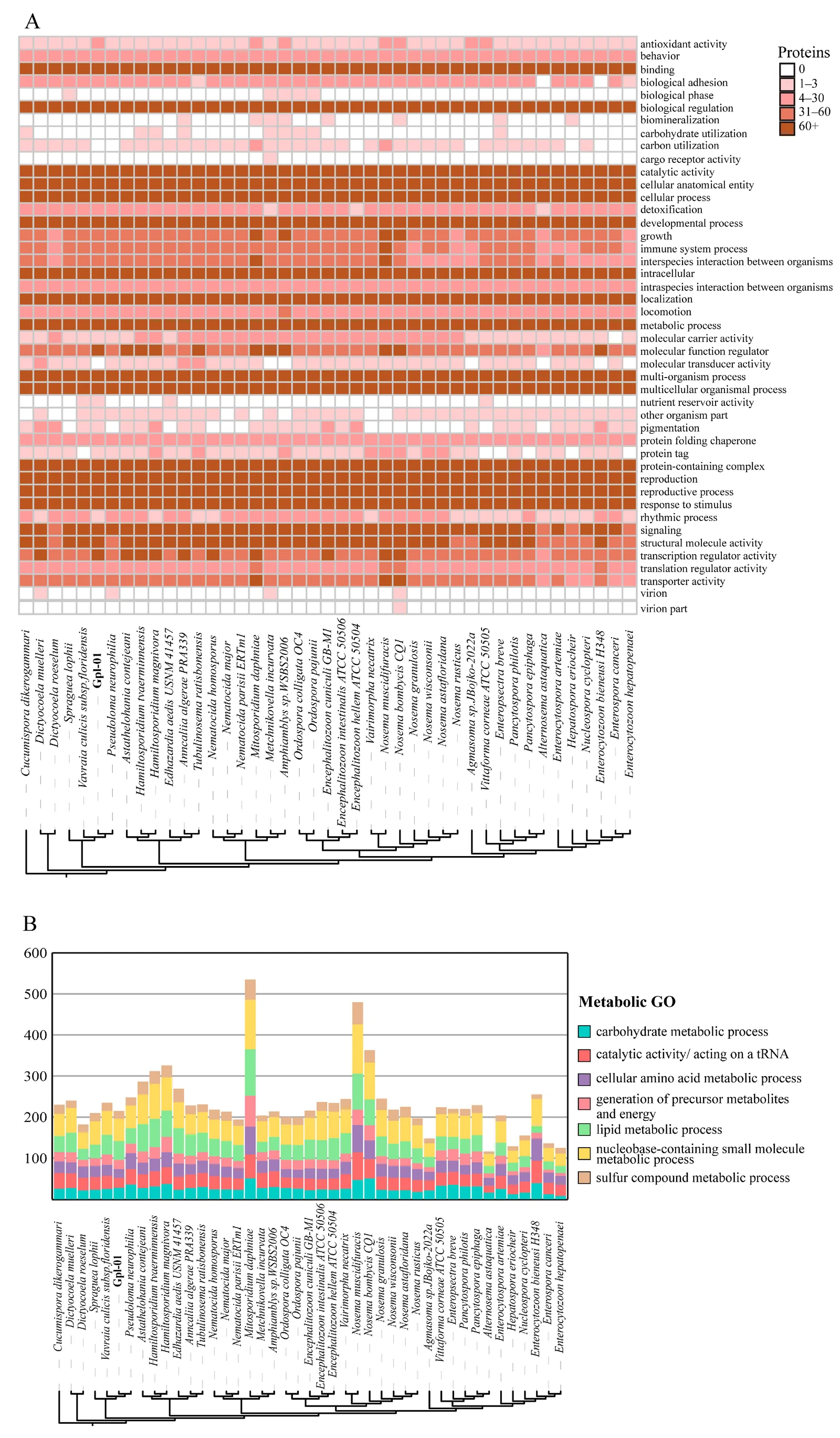
Comparative Gene Ontology functional profiles of microsporidian proteomes. (**A**) Heatmap showing the numbers of proteins assigned to GO Level 2 functional categories in *Glugea plecoglossi* and 42 other microsporidian taxa. White indicates no annotated proteins, whereas progressively darker shading represents 1–3, 4–30, 31–60, and >60 annotated proteins. (**B**) Stacked bar plot showing the numbers of proteins assigned to seven metabolism-related GO categories. Taxa in both panels are arranged according to the STAG species-tree topology shown in Figure 2. Because individual proteins may be assigned to multiple GO categories, the stacked bar heights in panel B represent summed annotation counts rather than numbers of nonredundant metabolism-related proteins.

Comparison of GO Level 2 profiles across the 43 microsporidian proteomes showed broadly similar distributions of the major functional categories (Figure 3A). Cellular processes, metabolic processes, binding, and catalytic activities contained relatively large numbers of annotated proteins in most taxa, whereas categories including carbohydrate utilization, biological adhesion, and carbon utilization were detected in fewer taxa. The overall GO profile of *G. plecoglossi* was broadly similar to that of its closely related taxa.

Comparison of the seven metabolism-related GO categories showed substantial variation in annotation counts among the 43 taxa (Figure 3B). Nucleobase-containing small molecule metabolic process was the most highly represented of the seven categories in nearly all taxa. In *G. plecoglossi*, annotation counts for cellular amino acid metabolic process and catalytic activity, acting on a tRNA were near the lower end of the cross-taxon distributions, whereas the other five categories occupied intermediate to relatively higher positions, indicating an uneven metabolism-related GO profile rather than a uniform shift across categories.

### 3.4. Comparative Repetitive-Sequence Composition of Microsporidian Genomes

**Figure 4:**
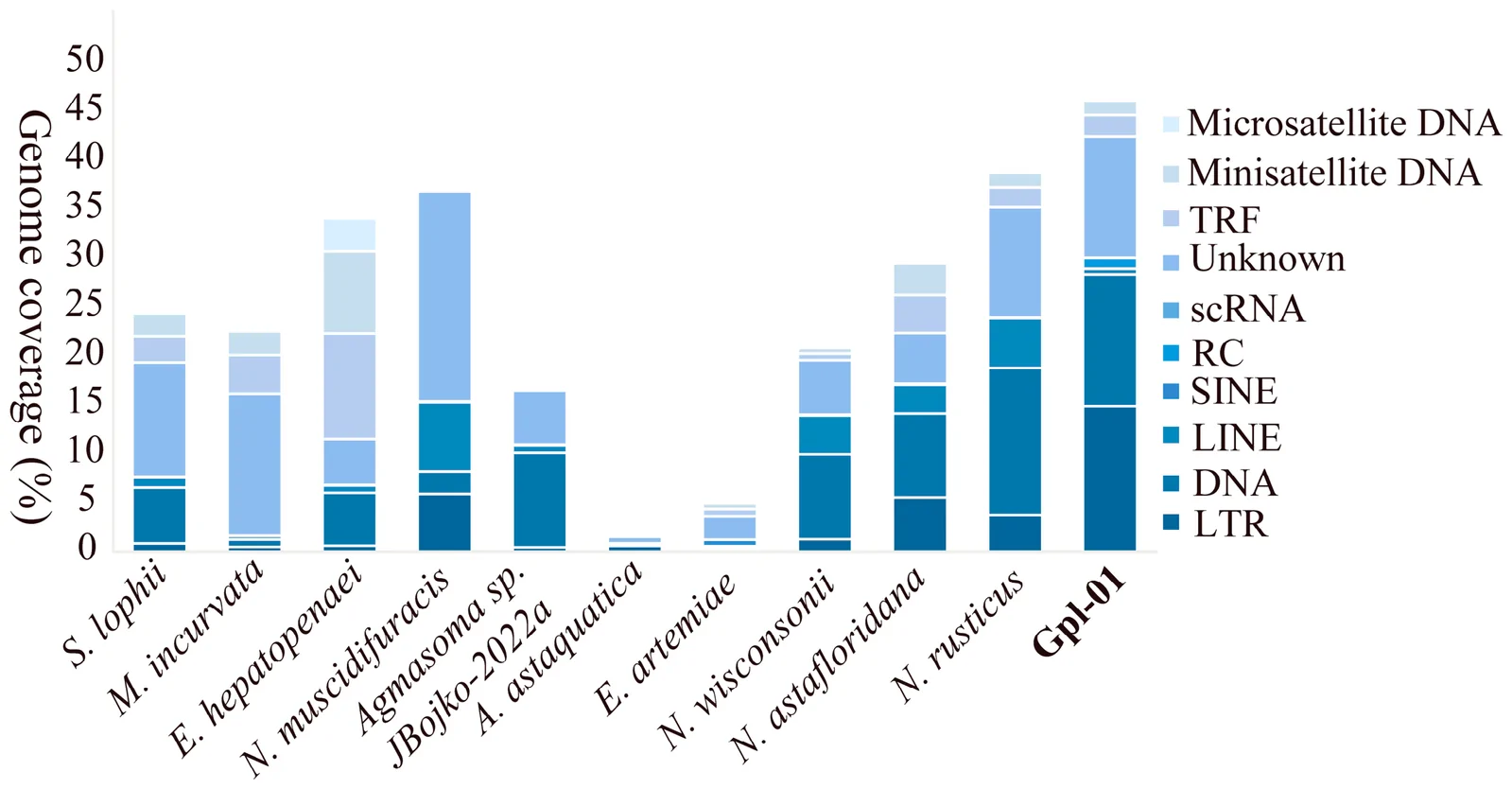
Comparative repetitive-sequence composition of *Glugea plecoglossi* and other microsporidian genomes. Stacked bars show the genomic coverage of individual repeat categories in *G. plecoglossi* Gpl-01 and 10 other microsporidian taxa. LTR, long terminal repeat retrotransposons; LINE, long interspersed nuclear elements; SINE, short interspersed nuclear elements; RC, rolling-circle elements; scRNA, scRNA-associated repeats; TRF, total tandem repeats identified using Tandem Repeats Finder. Minisatellite and microsatellite DNA represent subsets of the TRF-identified tandem repeats. Because different repeat categories may overlap and tandem-repeat subsets are displayed together with total TRF coverage, total bar height represents the arithmetic sum of the displayed category coverages and should not be interpreted as nonredundant total repetitive-sequence coverage. Gpl-01 is highlighted in bold to indicate the genome analyzed in this study.

Repetitive-sequence profiles differed substantially among the 11 microsporidian genomes examined (Figure 4). In *G. plecoglossi*, LTR elements, DNA transposons, and unclassified repeats showed genomic coverages of 14.86%, 13.49%, and 12.35%, respectively. LTR coverage was higher in *G. plecoglossi* than in the other taxa examined. By comparison, *Nosema muscidifuracis* showed relatively high contributions from unclassified repeats and LINEs, whereas DNA transposons represented a prominent repeat component in *Nosema rusticus*. *Enterocytospora artemiae* showed comparatively low coverage across most interspersed-repeat categories.

Tandem-repeat coverage also varied among the species examined (Figure 4). *Enterocytozoon hepatopenaei* had the highest total tandem-repeat coverage (10.82%), with minisatellite and microsatellite DNA accounting for 8.37% and 3.36%, respectively. Total tandem-repeat coverage was 3.96% in *Metchnikovella incurvata* and 3.85% in *Nosema astafloridana*. In *G. plecoglossi*, tandem repeats covered 2.24% of the genome assembly, including 1.49% minisatellite DNA and 0.04% microsatellite DNA.

### 3.5. Distribution and Protein Features of Multi-Copy Orthogroups

**Figure 5:**
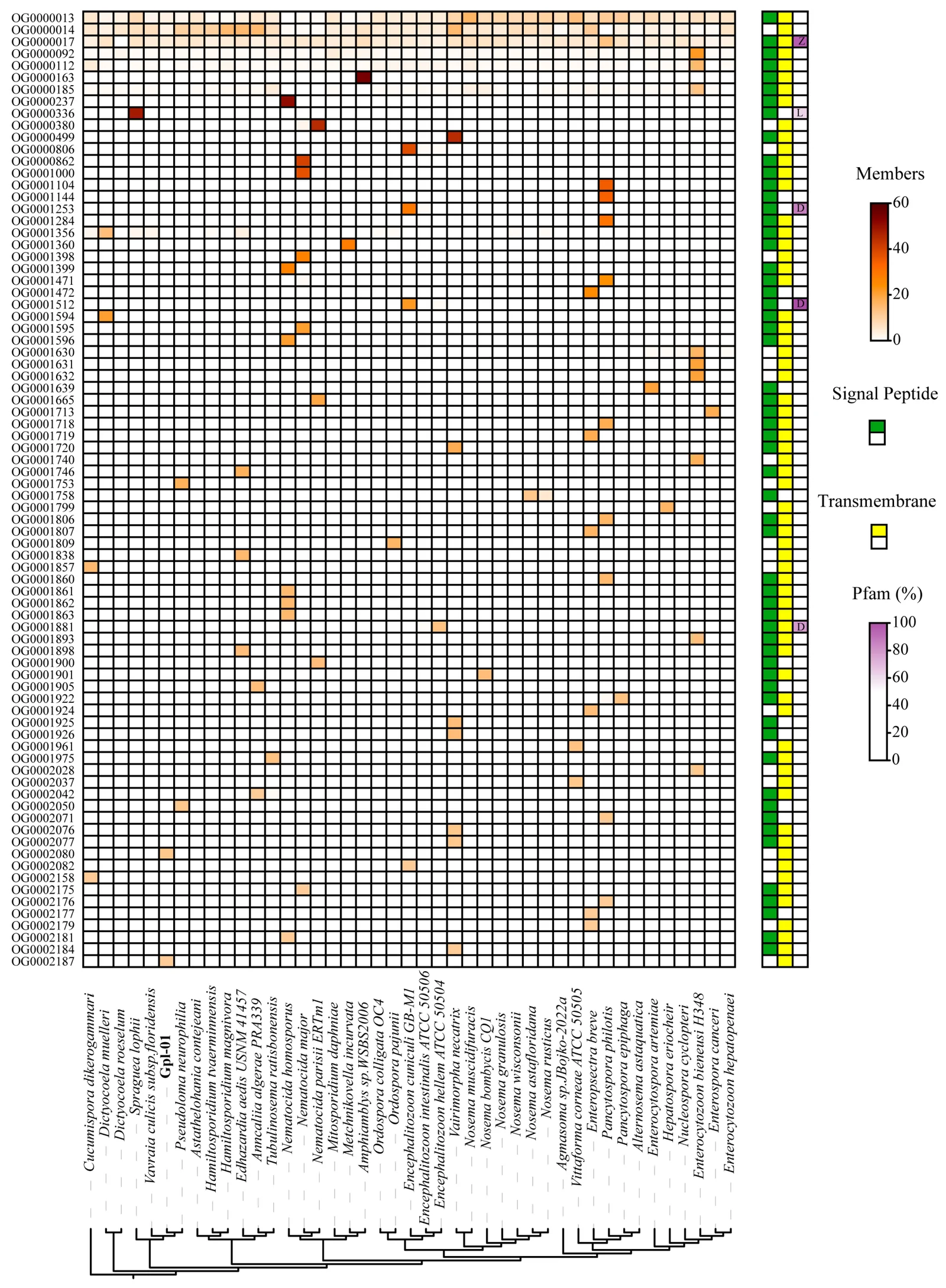
Distribution and protein features of multi-copy orthogroups across microsporidian taxa. The heatmap shows protein-member numbers of selected multi-copy orthogroups across 43 microsporidian taxa, with color intensity indicating member number. Taxa are arranged according to the species-tree topology shown in Figure 2. Annotations on the right indicate orthogroups in which at least 50% of member proteins were predicted to contain signal peptides or transmembrane domains, together with the proportions of selected Pfam domains. Letter codes indicate selected Pfam domain classes, where present: Z, zinc-finger; L, leucine-rich repeat (LRR); and D, DUF1609.

A total of 415 candidate multi-copy orthogroups were identified, each containing at least 10 members in at least one of the 43 taxa. Following selection of candidate multi-copy orthogroups and further filtering based on predicted signal peptides or transmembrane domains, the retained orthogroups showed strongly heterogeneous member distributions across taxa (Figure 5). High member numbers were generally concentrated in individual taxa or small groups of taxa, whereas the same orthogroups contained few or no members in most other species. The orthogroups with high member numbers also differed among taxa. In *G. plecoglossi* Gpl-01, relatively high member numbers were restricted to a limited subset of the displayed orthogroups, most notably OG0002080 and OG0002187, which contained 12 and 11 members, respectively, and were not represented in the other taxa examined.

## 4. Discussion

### 4.1. Genome Characteristics and Genomic Variation of Glugea plecoglossi

The present study establishes a genome resource for *G. plecoglossi* and places this fish-infecting microsporidian within a broad comparative genomic framework. The final 16.71 Mb scaffold-level assembly was consistent in overall scale with the genome size estimated by 21-mer analysis and contained 78.5% complete BUSCOs. Compared with the highly compact genomes characteristic of *Encephalitozoon* and several other canonical microsporidia, the *G. plecoglossi* genome lies toward the larger end of the distribution represented in our comparative dataset. This observation reinforces the view that extensive genomic reduction is a defining feature of microsporidian evolution but does not produce a uniform genome architecture across lineages [1,5,6,7,44,45]. Genome-size differences among microsporidia can reflect variation in gene density, non-coding sequence content, repetitive elements, and lineage-specific changes in gene content rather than a simple continuum of reductive evolution.

The 21-mer analysis also revealed substantial sequence heterogeneity in the pooled *G. plecoglossi* material, with a model-derived within-sample sequence variation of 2.68%. Levels and patterns of genomic variation differ among microsporidian species; genomic polymorphism has been documented in the fish parasite *Pseudoloma neurophilia*, whereas *E. cuniculi* exhibits extremely low heterozygosity [46,47]. In the present study, however, xenomas from multiple naturally infected fish were pooled before sequencing. The observed variation may therefore contain both allelic differences within parasite genomes and genetic differences among parasite individuals or genotypes. Accordingly, the 2.68% estimate should not be interpreted as the heterozygosity of a single *G. plecoglossi* genome. Isolate-level sequencing will be required to resolve the population structure and ploidy-related components underlying this variation. Because pooled parasite material can retain divergent haplotypes or genotype-specific sequence variants as separate assembled regions, some degree of assembly redundancy cannot be excluded. However, the close agreement between the final assembly size (16.71 Mb) and the 21-mer-based genome-size estimate (~16.1 Mb) argues against substantial inflation of the overall genome size, although more limited effects on genome-size and gene-copy comparisons remain possible.

### 4.2. Repetitive Sequences and Genome Architecture

The comparative repeat analysis revealed marked differences in repeat composition among the 11 microsporidian genomes examined. Previous studies have shown that the large variation in microsporidian genome size is often associated with differences in non-coding DNA, gene density, and transposable-element abundance [1,44,45,48,49,50]. In the uniformly analyzed subset of 11 genomes, *G. plecoglossi* showed particularly high coverage by LTR elements and substantial coverage by DNA transposons and unclassified repeats, whereas tandem-repeat coverage was more moderate. The repeat profile therefore differed from that of several other taxa rather than showing a uniform increase across all repeat classes.

This pattern is consistent with previous observations that repetitive elements can constitute an important component of relatively large microsporidian genomes. Diverse transposable elements have been identified in *Trachipleistophora hominis*, and transposable-element proliferation and gene duplication have also been associated with genome expansion in *Nosema bombycis* [7,48,50]. Nevertheless, the present comparison does not establish that repetitive-sequence accumulation caused the relatively large genome of *G. plecoglossi*. Because repetitive regions are intrinsically difficult to resolve during genome assembly, differences in assembly characteristics among genomes may influence the quantitative estimates of repeat coverage and should be considered when interpreting cross-species comparisons. Repeat abundance itself can additionally be shaped by lineage history, population-genetic processes, transmission mode, and the efficiency of selection against repetitive DNA [49]. The high LTR and DNA-transposon coverage observed here should therefore be considered a characteristic of *G. plecoglossi* genome architecture rather than direct evidence for a particular mechanism of genome expansion. Analysis of repeat age, insertion distribution, and transcriptional activity will be needed to determine how these elements have contributed to the evolutionary history of the genome.

### 4.3. Core Functional Similarity and Variation in Metabolism-Related Repertoires

Despite substantial differences in genome size and predicted protein number, the 43 microsporidian proteomes showed broadly similar distributions across major GO functional categories. Cellular process, metabolic process, binding, and catalytic activity remained prominent across much of the phylogeny, including in taxa with highly compact genomes. This broad functional similarity accords with comparative genomic evidence that extensive ancestral gene loss in microsporidia was accompanied by preferential retention of proteins required for essential cellular functions [1,6,8]. Importantly, because the GO comparisons were based on raw annotation counts without normalization, higher category counts should not be equated with increased functional complexity. Differences in proteome size and annotation coverage among taxa may contribute directly to these count-based comparisons.

The metabolism-focused comparison revealed greater heterogeneity beneath this broad functional similarity. In *G. plecoglossi*, annotation counts for cellular amino acid metabolic process and catalytic activity, acting on a tRNA were near the lower end of the respective cross-taxon distributions, whereas the other metabolism-related categories did not show similarly low relative representation. Thus, the relatively large *G. plecoglossi* genome was not accompanied by a uniform increase across metabolism-related GO categories. Such heterogeneity is consistent with the broader observation that individual metabolic functions differ substantially among microsporidian lineages. For example, *T. hominis* retains glycolytic and pentose-phosphate functions despite extensive metabolic reduction, whereas *E. bieneusi* has lost much of its core carbon metabolism and further loss of glycolytic functions characterizes the Enterocytozoonidae [7,9,10]. Recent comparisons of nematode-infecting microsporidia likewise show that broad conservation of core cellular functions can coexist with differences in individual metabolic pathways [8].

The relatively large genome of *G. plecoglossi* therefore does not appear to correspond to a proportional broadening of metabolism-related functional representation. Instead, its functional profile is more consistent with preservation of a common core of cellular functions accompanied by uneven representation of individual metabolism-related categories. Such a pattern is compatible with the broader microsporidian strategy of reducing endogenous biosynthetic capabilities while exploiting host-derived metabolites and energy [51].

### 4.4. Phylogenetic Position and Genomic Divergence Among Related Microsporidia

The phylogenomic analysis recovered *G. plecoglossi* as the sister taxon to the fish-infecting microsporidian *P. neurophilia*. The *G. plecoglossi–P. neurophilia* clade grouped with *V. culicis* subsp. *floridensis* and, in turn, with *S. lophii*. Genome-scale approaches are particularly valuable in microsporidia because high evolutionary rates and extensive sequence divergence can complicate phylogenetic inference based on individual markers [1]. The broader placement of this clade is consistent with previous genomic analyses showing *P. neurophilia* closely related to *V. culicis* and *S. lophii* [46].

Phylogenetic proximity, however, was not accompanied by similarity in genome size or coding capacity. The genomes of *P. neurophilia*, *V. culicis* subsp. *floridensis*, and *S. lophii* were all substantially smaller than that of *G. plecoglossi*. Related microsporidian lineages can experience markedly different genome histories, including independent changes in repetitive DNA, gene loss, duplication, and pseudogenization. For example, whole-genome duplication followed by subsequent genomic differentiation has been reported among *Spraguea* populations [52]. The evolutionary processes underlying the relatively large genome of *G. plecoglossi* remain unclear; nevertheless, these observations indicate that its genome size cannot be explained simply by its phylogenetic position [53].

The distribution of the morphological and molecular characteristics examined here further illustrates this distinction. The polar tube was broadly retained, whereas mitochondrial, regulatory, RNA-processing, and metabolic features showed heterogeneous distributions. Wadi et al. similarly showed that closely related nematode-infecting microsporidia can differ markedly in host range, tissue tropism, spore traits, and polar-tube characteristics while retaining many core cellular functions [8]. Thus, phylogeny defines the appropriate comparative context but does not by itself predict the retention of individual cellular traits.

This consideration is particularly relevant to the clade containing *G. plecoglossi*. Both *P. neurophilia* and *S. lophii* infect fish, whereas *V. culicis* primarily infects mosquitoes, so the topology does not support interpreting this group simply as a fish-adapted lineage. The capacity of *S. lophii* and *G. plecoglossi* to form prominent xenoma-like infection structures nevertheless provides an interesting comparative context. The *S. lophii* genome contains lineage-specific protein families and candidate secreted proteins that have been proposed to participate in host–parasite interactions [54]. This does not establish a shared molecular mechanism of xenoma formation, but it identifies comparative analysis of proteins potentially involved in host–parasite interactions within this phylogenetic neighborhood as a useful direction for future investigation.

### 4.5. Lineage-Structured Multi-Copy Orthogroups and Potential Roles at the Host–Parasite Interface

The multi-copy orthogroup analysis revealed a strongly lineage-structured pattern. Orthogroups with high member numbers were generally concentrated in individual taxa or restricted groups of species rather than being uniformly increased across microsporidia. This pattern is consistent with previous comparative analyses showing that extensive ancestral gene loss in microsporidia has been followed by lineage-specific emergence, duplication, or diversification of particular protein families [6,8]. Large species- and genus-specific families therefore represent an important component of microsporidian genome evolution even in the context of overall genomic reduction.

Several previously characterized large microsporidian families contain proteins positioned at the host–parasite interface. Host-exposed proteins in *Nematocida* are enriched in rapidly evolving, lineage-specific families [55], while expanded LRR and Ricin B lectin-like families have been described in *S. lophii* [54]. Gene-family multiplication can also contribute directly to host-resource exploitation; multiple nucleotide transporter paralogs in *T. hominis*, for example, participate in acquisition of host-derived purine nucleotides [56]. These examples illustrate that lineage-specific increases in family membership may affect diverse aspects of intracellular parasitism.

In *G. plecoglossi*, relatively high member numbers were confined to a limited subset of the orthogroups displayed in Figure 5 rather than occurring broadly across many candidate families. Most notably, OG0002080 and OG0002187 contained 12 and 11 members, respectively, and were detected only in *G. plecoglossi* within the present comparative dataset. Both orthogroups met the transmembrane-domain selection criterion but lacked recognizable Pfam domains in the present annotation. Their lineage-restricted distribution, relatively high member numbers, and predicted membrane association make them of particular interest for further characterization. However, these patterns should be regarded as candidates for lineage-associated diversification rather than evidence of demonstrated gene-family expansion, and their biological functions remain unknown.

Several aspects of the present analysis should nevertheless be interpreted with caution. Presence/absence assignments of morphological and molecular characteristics depend on genome assembly completeness, available genome annotations, and published evidence, and therefore may remain uncertain when direct functional evidence is lacking. Cross-taxon comparisons of predicted gene numbers and GO annotation counts may also be influenced by differences in assembly completeness and gene-prediction or annotation quality among taxa. Given the sequence heterogeneity and potential assembly redundancy associated with the pooled parasite material, the 19.8% duplicated BUSCOs may reflect, at least in part, unresolved allelic or genotype-level differences retained as separate assembled regions. However, genuine biological duplication cannot be distinguished from assembly redundancy on the basis of the present data. Multi-copy orthogroup patterns should therefore be interpreted cautiously, particularly for orthogroups showing relatively high member numbers only in *G. plecoglossi*, such as OG0002080 and OG0002187. In addition, the multi-copy orthogroup analysis was intended to identify conspicuous differences in family membership rather than to reconstruct ancestral family sizes or formally test gene-family expansion and contraction.

## 5. Conclusions

This study establishes a genome resource for *G. plecoglossi* and defines its position within a broad comparative framework of microsporidian genomes. *G. plecoglossi* possesses a relatively large genome characterized by substantial LTR and DNA-transposon coverage, while showing broad similarity to other microsporidia in major GO functional profiles and uneven representation among metabolism-related functional categories. Its close phylogenetic relationship to *P. neurophilia*, *V. culicis* subsp. *floridensis*, and *S. lophii* contrasts with substantial differences in genome size and multi-copy orthogroup distributions among these taxa. These data expand the limited genomic resources available for fish-infecting microsporidia and provide a foundation for future studies of parasite development, host interaction, and xenoma-associated biology.

## Figures and Tables

**Table 1:** Genome assembly and annotation statistics of *Glugea plecoglossi*.

Category	Parameter	Value
Genome assembly	Assembly size	16,711,591 bp
	Number of scaffolds	59
	Mean scaffold length	283,247 bp
	Scaffold N50	557,527 bp
	Scaffold N90	115,546 bp
	Maximum scaffold length	1,072,421 bp
	Minimum scaffold length (bp)	33,497 bp
	GC content	31.42%
	N content	0%
Gene prediction	Predicted gene loci	4331
	Gene models with corresponding mRNA/CDS/protein sequences	4324
	Mean predicted CDS length	1262 bp
	Mean predicted protein length	~420 aa
Genome annotation	Genes annotated in ≥1 functional database	2786
	Functional annotation rate	64.43%
ncRNA	Predicted ncRNAs	98
Completeness	Complete BUSCOs	78.5%

Note: A total of 4331 protein-coding gene loci were predicted, of which 4324 had corresponding mRNA, CDS, and protein sequences and were used for downstream protein functional annotation. The functional annotation rate represents the proportion of these 4324 predicted proteins annotated in at least one of the NR, Swiss-Prot, eggNOG/KOG, GO, KEGG, or CAZy databases. Genome completeness was assessed using BUSCO v6.0.0 against the microsporidia_odb10 lineage dataset comprising 600 BUSCO groups.

## Data Availability

The genome assembly of *Glugea plecoglossi* Gpl-01 has been deposited in DDBJ/ENA/GenBank under accession JCDPZV000000000 (version JCDPZV010000000), associated with BioProject PRJNA1523027 and BioSample SAMN63022018. Accession numbers for the publicly available microsporidian genomes used in the comparative analyses are provided in Table S4. Additional data supporting this study are available in the article and its Supplementary Materials.

## Supplementary Materials

The following supporting information can be downloaded at: https://www.mdpi.com/article/10.3390/biology15201848/s1, Figure S1: Sequencing data quality and genome survey of *Glugea plecoglossi*; Figure S2: Structural characteristics of predicted protein-coding genes in *Glugea plecoglossi*; Table S1: Sequencing statistics and genome survey parameters of *Glugea plecoglossi*; Table S2: Detailed protein-coding gene and ncRNA annotation of *Glugea plecoglossi*; Table S3: Functional annotation summary of protein-coding genes in *Glugea plecoglossi*; Table S4: Genome assembly information and host characteristics of microsporidian taxa included in the comparative genomic analysis.
