# Supplementary material for: Comparative Genomic Analysis Reveals Evolutionary Features of Glugea plecoglossi, a Fish-Infecting Microsporidian Parasite of Ayu (Plecoglossus altivelis)
Source: Biology (Basel). 2026 Oct 15;15(20):1848. doi: 10.3390/biology15201848 (PMC13702014; doi:10.3390/biology15201848)
Supplement: Supplementary file 1 [file biology-15-01848-s001.zip › Supplementary Figure S1. Sequencing data quality and genome survey of Glugea plecoglossi.pdf]

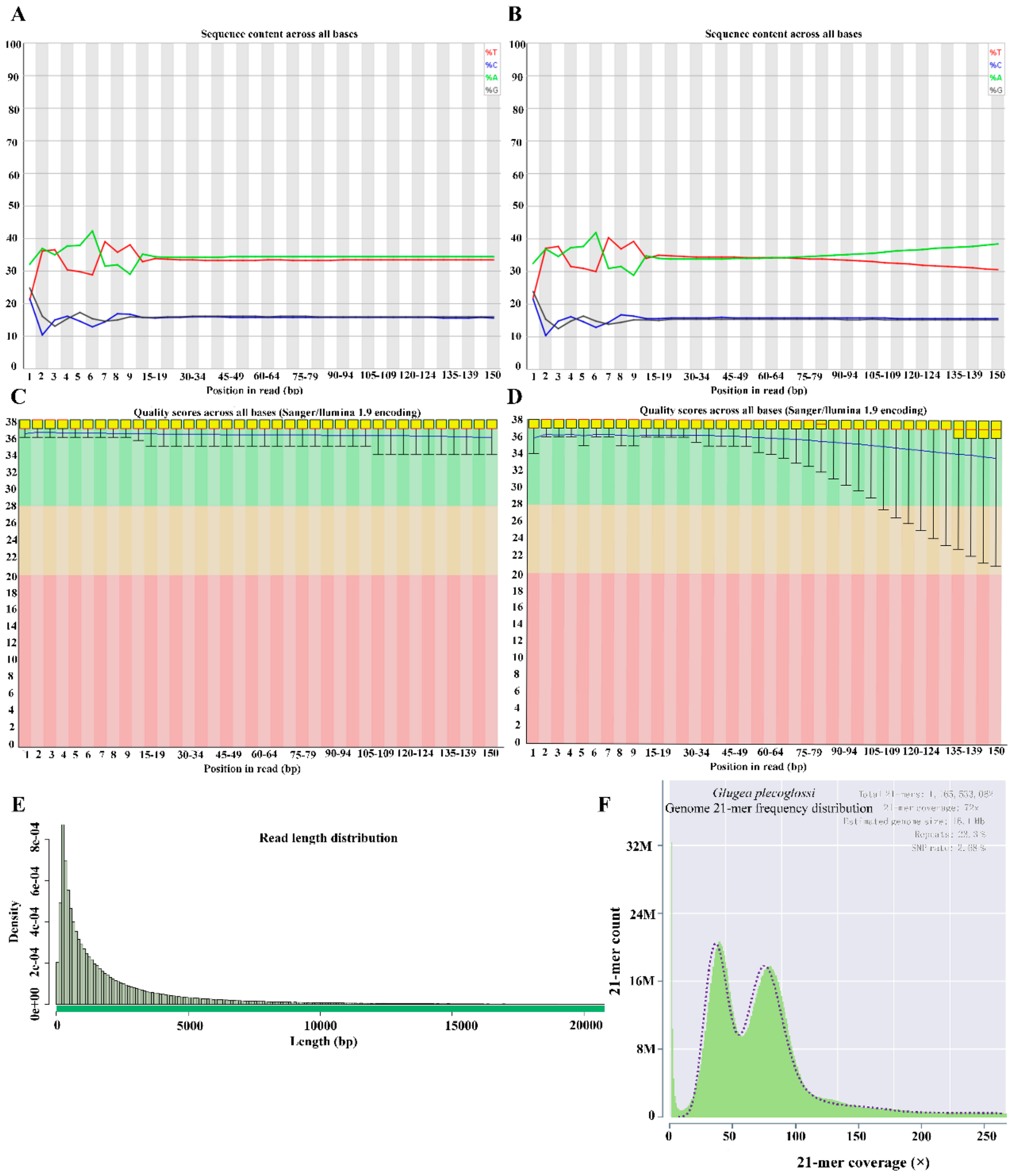

Supplementary Figure S1. Sequencing data quality and genome survey of *Glugea plecoglossi*.

(A, B) Per-base nucleotide composition of Illumina R1 and R2 reads, respectively; colored curves indicate the proportions of A, T, C, and G at each sequencing position. **(C, D)** Per-base quality-score distributions of R1 and R2 reads, respectively. The blue line indicates the mean quality score, yellow boxes represent the interquartile range, the red line denotes the median, and whiskers indicate the 10th–90th percentile range; green, orange, and red backgrounds denote high-, moderate-, and low-quality regions, respectively. **(E)** Read-length distribution of third-generation sequencing reads, showing a right-skewed distribution with a long tail. **(F)** 21-mer frequency distribution used for genome survey analysis. The x-axis represents 21-mer coverage depth and the y-axis the corresponding 21-mer count; genome size, repeat content, and within-sample

sequence variation were estimated using the JCVI 21-mer model.
