# Supplementary material for: Comparative Genomic Analysis Reveals Evolutionary Features of Glugea plecoglossi, a Fish-Infecting Microsporidian Parasite of Ayu (Plecoglossus altivelis)
Source: Biology (Basel). 2026 Oct 15;15(20):1848. doi: 10.3390/biology15201848 (PMC13702014; doi:10.3390/biology15201848)
Supplement: Supplementary file 1 [file biology-15-01848-s001.zip › Supplementary Figure S2. Structural characteristics of predicted protein-coding genes-R1.pdf]

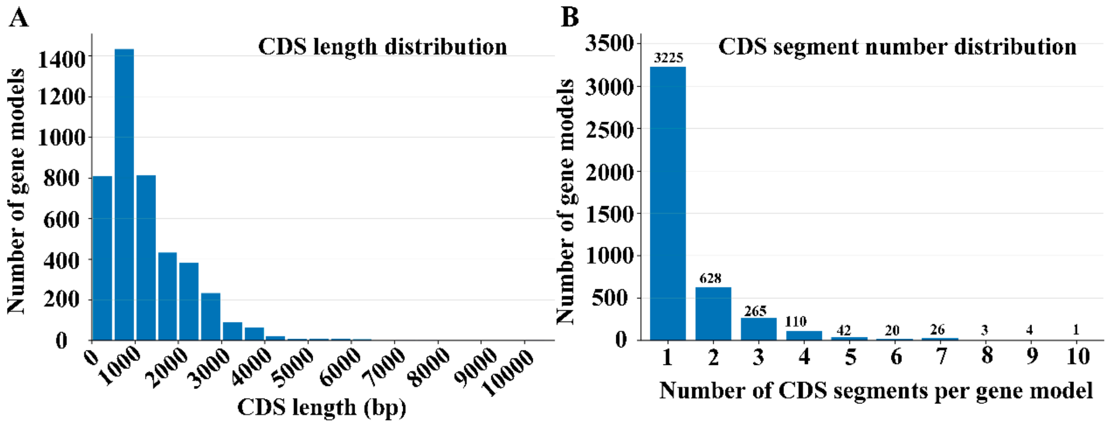

Supplementary Figure S2. Structural characteristics of predicted protein-coding genes in *Glugea plecoglossi*.

(A) Distribution of CDS lengths among 4,324 protein-coding gene models, using a bin width of 500 bp. **(B)** Distribution of the number of CDS segments per protein-coding gene model. Because exon features were not separately annotated in the GFF file, discontinuous CDS features assigned to the same mRNA were counted as separate CDS segments for this summary.
