# Supplementary material for: Comparative Genomic Analysis Reveals Evolutionary Features of Glugea plecoglossi, a Fish-Infecting Microsporidian Parasite of Ayu (Plecoglossus altivelis)
Source: Biology (Basel). 2026 Oct 15;15(20):1848. doi: 10.3390/biology15201848 (PMC13702014; doi:10.3390/biology15201848)
Supplement: Supplementary file 1 [file biology-15-01848-s001.zip › Supplementary Table S3 Functional annotation summary of protein-coding genes-R1.pdf]

Table S3. Functional annotation summary of protein-coding genes in *Glugea plecoglossi*.

**Annotation database or category**	**Annotated genes**	**Percentage of predicted proteins (%)**
NR	2,781	64.32
eggNOG/KOG	1,774	41.03
Swiss-Prot	1,375	31.80
GO	883	20.42
KEGG	658	15.22
CAZy	24	0.56
Annotated in at least one database	2,786	64.43
Not annotated in any database	1,538	35.57

Note: A single gene may be annotated by multiple databases; therefore, annotation counts from different databases are not additive. "Annotated in at least one database" represents the non-redundant union of gene identifiers across all annotation databases.
